# The Genome of the Steller Sea Lion (*Eumetopias jubatus*)

**DOI:** 10.3390/genes10070486

**Published:** 2019-06-26

**Authors:** Harwood H. Kwan, Luka Culibrk, Gregory A. Taylor, Sreeja Leelakumari, Ryan Tan, Shaun D. Jackman, Kane Tse, Tina MacLeod, Dean Cheng, Eric Chuah, Heather Kirk, Pawan Pandoh, Rebecca Carlsen, Yongjun Zhao, Andrew J. Mungall, Richard Moore, Inanc Birol, Marco A. Marra, David A.S. Rosen, Martin Haulena, Steven J. M. Jones

**Affiliations:** 1 Canada’s Michael Smith Genome Sciences Centre, British Columbia Cancer, Vancouver, BC V5Z-4S6, Canada; 2 Department of Medical Genetics, University of British Columbia, Vancouver, BC V6T-1Z4, Canada; 3 Department of Graduate Studies, Bioinformatics, University of British Columbia, Vancouver, BC V6T-1Z4, Canada; 4 Institute for the Oceans and Fisheries, University of British Columbia, Vancouver, BC V6T-1Z4, Canada; 5 Vancouver Aquarium, Vancouver, BC V6G 3E2, Canada; 6 Department of Molecular Biology and Biochemistry, Simon Fraser University, Burnaby, BC V5A-1S6, Canada

**Keywords:** Steller sea lion, *Eumetopias jubatus*, genome, microfluidic partitioning, nanopore, marine animal

## Abstract

The Steller sea lion is the largest member of the Otariidae family and is found in the coastal waters of the northern Pacific Rim. Here, we present the Steller sea lion genome, determined through DNA sequencing approaches that utilized microfluidic partitioning library construction, as well as nanopore technologies. These methods constructed a highly contiguous assembly with a scaffold N50 length of over 14 megabases, a contig N50 length of over 242 kilobases and a total length of 2.404 gigabases. As a measure of completeness, 95.1% of 4104 highly conserved mammalian genes were found to be complete within the assembly. Further annotation identified 19,668 protein coding genes. The assembled genome sequence and underlying sequence data can be found at the National Center for Biotechnology Information (NCBI) under the BioProject accession number PRJNA475770.

## 1. Introduction

Steller sea lions (*Eumetopias jubatus*) inhabit the coastal waters of the subarctic and are mainly found in the northern Pacific Rim, stretching from central California to northern Japan [1]. Genetic analyses have identified three distinct Steller sea lion populations: the eastern, western and Asian populations [2,3]. The Steller sea lion is the largest member of the sea lion and fur seal family, Otariidae [1,4], and is only supplanted in size by the walrus and the northern and southern elephant seal amongst pinnipeds. Similar to other otariids, the Steller sea lion is amphibious, hauling-out onto land to reproduce, pup rear, molt and rest, while their time in the water is spent feeding, with a diet consisting of a wide range of fish as well as cephalopods [5,6]. Despite being top-level carnivores, Steller sea lions are still susceptible to predation, primarily by larger aquatic species such as killer whales (orcas) and sharks [7]. 

Significant population declines in the 1970s and 1980s eliminated more than 80% of the Steller sea lion population, leading to the species being listed as “threatened”, with the western regional populations being reclassified “endangered” in 1997 [1]. Although the exact reason for the population decline has yet to be defined, many suspected causes have been identified; including overfishing of fish stock in the Gulf of Alaska; increased predation by orcas and sharks; indirect effects of climate changes; the effects of diseases, parasites and contaminants; as well as hunting by humans [8,9,10]. 

Recent research has explored the genetic basis of mammalian adaptation into the marine environment, unveiling a multitude of genes which have accelerated evolutionary rates in marine mammalian lineages [11]. These genes appear to be enriched in pathways that control many functional adaptations for marine animals, including sensory systems, muscle physiology and lipid metabolism [11]. An increase in mammalian species with complete genetic information may aide in the progression and affirmation of these studies. 

Here we present the genomic sequence and gene annotation resources for the Steller sea lion. This assembly will assist in the conservation process of Steller sea lions, as well as contribute to the comparative genomic analysis of marine mammals, aiding in our understanding of how marine animals may have adapted in their transition back to the water.

## 2. Materials and Methods 

The genome was assembled from a 10x Genomics Chromium microfluidic partitioned genomic DNA (gDNA) library and was subsequently modified and scaffolded using a nanopore library. Sequencing was performed using an Illumina HiSeqX (Illumina, San Diego, CA, USA) instrument and a MinION sequencer (Oxford Nanopore Technologies (ONT), Oxford, UK) at Canada’s Michael Smith Genome Sciences Centre, BC Cancer. The animal under study was a female Steller sea lion (ISIS/GAN: 26980396). She was born in the wild in British Columbia, Canada in 2000 and brought to the aquarium a pup. She has lived at the Vancouver Aquarium ever since. The Vancouver Aquarium is accredited by the American Association of Zoos and Aquariums (AZA). Peripheral blood was extracted as part of a routine veterinary preventative health care plan performed as part of the AZA credentialing requirements. Surplus blood was used for DNA sequencing. 

The microfluidic partitioned library was constructed as follows: High molecular weight (HMW) gDNA was extracted from the peripheral blood with the QIAGEN MagAttract HMW DNA Kit (QIAGEN, Germantown, MD, USA), using the HMW gDNA extraction protocol as detailed in the Chromium Genome Reagent Kits Version 2 User Guide (PN-120229) (10x Genomics, Pleasanton, CA, USA). Quality of the gDNA was then assessed using pulse-field gel electrophoresis (PFGE). Gel Bead-In-Emulsions (GEMs) were then created from a library of Genome Gel Beads combined with 1 ng of gDNA, Master Mix, and partitioning oil, using the 10x Genomics Chromium Controller instrument with a micro-fluidic Genome chip (PN-120216). The GEMs were then subjected to an isothermic amplification step. Bar-coded DNA fragments were extracted and underwent Illumina library construction, as detailed in the Chromium Genome Reagent Kits Version 2 User Guide (PN-120229). Library yield was measured through quantitative PCR (qPCR). Library fragment size and distribution was measured using an Agilent 2100 Bioanalyzer DNA 1000 chip (Santa Clara, CA, USA), with 500 bp fragments being the goal. The library was then run on an Illumina HiSeqX sequencer (Illumina) with a paired-end protocol to produce 150 bp reads for downstream genome construction. This resulted in 797.9 million 150 bp reads, which corresponded to an estimated 34.65-fold genome coverage. 56.02% of the reads were nonduplicates and could be phased, while 10.63% of the reads were duplicates. The estimated weighted mean molecule length for the library was 38.03 Kb.

The nanopore library was constructed as follows: high-quality gDNA was extracted as previously described. The gDNA was subjected to size selection and sequencing preparation using the Library Loading Bead Kit R9 (ONT) (EXP-LLB001). Adapters were added using the Ligation Sequencing Kit 1DR9 (ONT) (SQK-LSK108). The MinION flow cell was prepared with the Flow Cell Priming Kit (ONT) (EXP-FLP001). The prepared gDNA was loaded onto the MinION flow cell and was run in the MinION sequencer to produce long reads. The sequencing resulted in around 877 thousand reads containing 9 billion sequenced bases, with an N50 of 22.7 Kbp, an approximately 4-fold genome coverage. 

Genome assembly was performed on the paired-end sequence reads from the partitioned library using Supernova (version 2.1.1, 10x Genomics, San Francisco, CA, USA). The initial Supernova assembly was 2.404 Gbp with a genomic scaffold N50 length of 41.76 Mbp and a contig N50 length of 174.4 Kbp (Table 1). The phase block N50 length of the initial Supernova assembly was 426.69 Kbp. Improvements to the initial assembly were made by correcting misassemblies, re-scaffolding through the incorporation of the nanopore long read data, and by gap filling.

Briefly, misassemblies in the genome were identified and corrected using Tigmint (version 1.1.2, Canada’s Michael Smith Genome Sciences Centre; parameters -span at 20; -window at 1000; -minsize at 2000; -as at 0.65; -nm at 5; -dist at 50,000; -mapq at 0; -trim at 0; -t at 8) [12]. Altogether, 625 cuts were made by Tigmint, rendering the N50 length to 13.55 Mbp. Scaffolding was then performed using the Assembly Roundup by Chromium Scaffolding algorithm (ARCS) (version 1.0.1 Canada’s Michael Smith Genome Sciences Centre; parameters -c at 5; -e at 30000; -r at 0.05) [13], which improves the contiguity of the assembly through organization using the 10x Genomics linked reads. Further scaffolding on the assembly was done with LINKS (version 1.8.5, Canada’s Michael Smith Genome Sciences Centre; parameters -d at 4000; -k at 15; -e at 0.1; -l at 5; -a at 0.3; -t at 5; -o at 0; -z at 500; p at 0.001; -x at 0) [14], using information from the uncorrected nanopore long reads to assist in the scaffolding. Altogether, the scaffolding steps improved the scaffold N50 length to 14.02 Mbp. Gaps were filled with Sealer (version 2.0.2, Canada’s Michael Smith Genome Sciences Centre; parameters -k at 90 to 120, step 10) [15], using the Illumina reads from the Chromium library to populate the Bloom filter. The nanopore reads were not utilized for Sealer as they have a high sequencing error rate relative to the Illumina reads. Altogether, Sealer closed a total of 6681 gaps, improving the contig N50 to 242.2 Kbp.

Benchmarking Universal Single-Copy Orthologs (BUSCO) [16], a program which attempts to reconstruct a set of conserved mammalian genes in a genome, was used as an assessment for genome completeness after every step of the assembly (Table 1). The reconstruction rate of BUSCO genes improved slightly after each stage of the assembly process, with 3904 genes identified in the assembly from a set of 4104 highly conserved mammalian genes. An additional 102 genes were present but in a fragmented state. 

The genome was annotated using the NCBI Eukaryotic Genome Annotation Pipeline and considered of sufficient quality to become a RefSeq reference genome [17]. Within the genome, 19,668 coding genes were identified, along with 3786 non-coding genes, 6814 pseudogenes, and 69 immunoglobulin/T-cell receptor gene segments. 

## 3. Results and Discussion

The final assembled *Eumetopias jubatus* genome consisted of 2,404,049,571 sequenced bases with a scaffold N50 of 14.02 Mbp, representing a good overview of the 18 chromosome pairs in the Steller sea lion [18]. Examination of the predicted Steller sea lion genes revealed that 25 of the top 100 frequently lost genes in marine mammals identified by Chikina et al. [11] were missing or were severely altered (by frameshift or truncation), resulting in a nonfunctional protein. The missing or altered genes were: *SSTR4*, *PDE1C*, *GRIN3B*, *PDEC1*, *LHFP*, *TMEM235*, *ASIC4*, *CYP3A34*, *PRSS3*, *FMO1*, *C1QTNF8*, *GSTM4*, *KRTAP12-3*, *CALML3*, *OR51I2*, *OR51I1*, *OR10Z1*, *OR5C1*, *OR4E2*, *OR6K6*, *OR4S1*, *OR51V1*, *OR52K1*, *OR10G3*, *OR1I1*. The Steller sea lion genome also contained a homozygous lesion in *PON1*, a gene found to have accrued deleterious lesions in all marine mammal lineages, while remaining functional in terrestrial mammals [19]. As the majority of these genes are olfactory and gustatory, their loss helps to solidify evidence that these genes are marine-accelerated, and that reductions in the sense of taste and smell are ubiquitous in marine mammals (despite their importance in social interactions) [11,20]. Additionally, the loss of *PON1* may be crucial in the future conservation of the species, due to the role the gene plays in mammals in the defense against neurotoxicity from specific man-made organophosphorus compounds [19].

The closest relative to the Steller sea lion with a sequenced genome is the California sea lion (*Zalophus californianus*) [21]. Both species contain a diploid karyotype with 18 chromosome pairs [18]. Alignment of the Steller sea lion assembly to the California sea lion assembly with the Burrows-Wheeler Aligner MEM algorithm (BWA-MEM)(version 0.7.17) [22] yielded a variant rate of 1 variant in every 183 bases, indicating a 99.5% similarity between the two genomes. An overview of assembly statistics of both genomes is presented in Table 2, where the Steller sea lion is shown to be more contiguous at the DNA level, while Dovetail Hi-C scaffolding of the California sea lion allowed for increased scaffolding. An examination with BUSCO on both genomes using the 4104 gene mammalian dataset suggests similar completeness in both genomes, with a slight advantage to the California sea lion.

**Figure 1:**
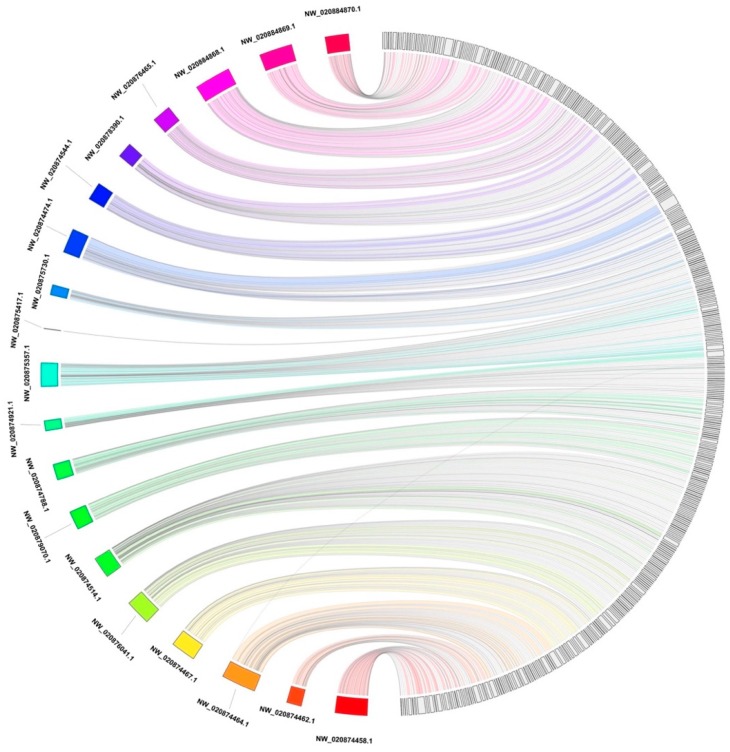
A Jupiter plot illustrating the global genome alignment of the Steller sea lion genome (**right**) to the California sea lion genome (**left**). Alignment was accomplished with BWA-MEM. Connections within the circle represent alignment between the two assemblies. California sea lion scaffolds over 10 Mb in length were selected. The longest Steller sea lion scaffolds which sum to the same amount of sequence were also selected. Only alignments over 10 Kb in length are displayed.

The alignment of the Steller sea lion assembly to the California sea lion assembly through BWA-MEM (version 0.7.17) [22] was subsequently visualized as a Jupiter plot (Figure 1) [23], a Circos [24] based genome assembly consistency plot used to view large scale translocations or other large structural variations. The connecting bands within the circle represent regions of synteny, whereas the blocks on the arc of the circle represent the largest scaffolds in the assembly. The lack of diagonal lines extending from the middle of the scaffold block suggests there are no definite breaks in the synteny between the two assemblies at 10 Kb resolution. 

Both assemblies have been annotated by the NCBI Eukaryotic Genome Annotation Pipeline [17]. A comparison of the annotation statistics is present in Table 3. While the California sea lion appears to have a higher identified gene count, the majority of this difference can be attributed to regions predicted to be non-coding genes. Variant calling using samtools [25] and snpEff [26] revealed 91,797 coding variant changes between the two species, with 33,860 missense variants and 44,597 synonymous variants. The ten genes containing the most variants are: *AHNAK2* (424 SNVs), a zinc-finger protein-like gene (386 SNVs), a basic proline-rich protein-like gene (239 SNVs), *THAP12* (211 SNVs), *VWA2* (200 SNVs), *AKAP17A* (154 SNVs), *PPFIA3* (173 SNVs), *FLG2* (230 SNVs), *GLYCTK* (189 SNVs), a *SPATA31D3*-like gene (159 SNVs), and *RCOR2* (155 SNVs). In-depth phylogenetic assessment will be needed to determine the genetic differences responsible for speciation between the two sea lions. 

The Steller sea lion assembly shows that microfluidic partitioned libraries greatly improve assembly of complete genomes. Additional information from nanopore reads is also shown to improve scaffolding, resulting in a high-quality reference genome from multiple sources of genomic sequence. A reference Steller sea lion genome may assist the understanding of the genetic effects of population decline, and ultimately aid in the conservation process. Additionally, the genome, alongside the California sea lion reference genome, can serve as a strong starting point for evolutionary studies regarding the divergence of sea lions from other pinnipeds.

## Figures and Tables

**Table 1:** Comparison of assembly statistics for steps in the assembly process.

Assembly	Total Size (Gbp)	No. of Gaps	Contig N50 (Kbp)	No of Scaffolds	Scaffold N50 (Mbp)	Longest Scaffold (Mbp)	BUSCO Complete Genes (of 4104)
Supernova	2.404	24,113	174.4	7238	41.76	146.1	95.0% (3899)
Tigmint	2.404	24,086	174.4	7748	13.55	59.02	95.0% (3901)
ARCS/LINKS	2.404	24,145	174.4	7689	14.02	59.02	95.1% (3903)
Sealer	2.404	17,464	242.4	7689	14.02	59.02	95.1% (3904)

**Table 2:** Assembly statistics of the Steller sea lion and the California sea lion.

Assembly	Total Size (Gbp)	No. of Contigs	Contig N50 (Kbp)	Contig L50	No of Scaffolds	Scaffold N50 (Mbp)	Scaffold L50	BUSCO Complete Genes (of 4104)
Steller sea lion	2.404	24,747	242.4	2995	7472	14.02	54	95.1% (3904)
California sea lion	2.367	57,871	97.7	7181	10,423	143.4	7	95.4% (3912)

**Table 3:** Annotation summary for the sea lion assemblies.

Assembly	Total Count	Protein Coding	Non-Coding	Pseudogenes	Immunoglobulin/ T-Cell Receptor Gene Segments
Steller sea lion	30,336	19,668	3786	6814	68
California sea lion	32,113	19,617	5644	6785	67
